# The current status and implications of aging research from a health perspective

**DOI:** 10.3389/fpsyg.2026.1775885

**Published:** 2026-05-21

**Authors:** Yaming Lu

**Affiliations:** Academy of Future Education, Xi'an Jiaotong-Liverpool University, Suzhou, China

**Keywords:** environmental support, healthy aging, intrinsic capacity, older adults, physical and mental health

## Abstract

**Background:**

With the pace of aging population deepening, the well-being of older adults has increasingly emerged as the focus of global concern. In this context, this study adopts a narrative review approach. Drawing on the concept of healthy aging and incorporated by Person–Environment Fit theory, an analytical framework is developed to systematically synthesize research on aging from a health perspective. It aims to explore the impacts of intrinsic capacity (including physical function, cognitive function and mental health), external environmental support (including social environment, physical environment and technology environment), and their interactive effects on healthy aging.

**Methods:**

To perform this narrative review, a literature search was conducted in the Chinese and international databases including CNKI, Wanfang, PubMed, ScienceDirect and Sage Journals databases. The search covered publications from database inception to December 31, 2025. Studies were screened and relevant information was retrieved in alignment with the predefined inclusion and exclusion criteria.

**Main findings:**

Evidence indicates that intrinsic capacity and external environmental support are interconnected. Environmental resources can compensate for declines in intrinsic capacity while also facilitating its maintenance and development. Conversely, higher levels of intrinsic capacity enable individuals to more effectively access and utilize environmental resources, thereby creating a positive feedback loop that promotes health.

**Conclusion:**

The interplay between intrinsic capacity and external environment support plays a decisive role in shaping health outcomes in later life and it is fundamental to achieving health aging. The mechanisms underlying their interaction and their combined impact on health should be examined in further research. There is also a need to develop and refine measurement tools for person–environment fit and strengthen cross-cultural comparative research.

## 1 Introduction

Data from the National Bureau of Statistics demonstrate that, by the end of 2025, individuals over 60 in China have attained 323 million, making up 23% of the overall population (National Bureau of Statistics, 2026). By 2035, the population aged 60 and above will exceed 400 million, and China will enter a “super-aged” society. With the rapid growth of the elderly population, the risks of chronic diseases, multimorbidity, and functional limitations, as well as demand for healthcare and long-term care services are increasing. Ensuring older adults maintain good physical-mental health and enjoy a healthy and fulfilling later life has become one of the major social development issues.

To address the aging population proactively, the World Health Organization has proposed the notion of healthy aging based on the life course theory. This framework emphasizes the promotion of functional ability in older adults, which is shaped by intrinsic capacity, environmental support, and interactions between the two. However, existing aging research has largely focused on the independent effects of intrinsic capacity or environmental factors, with limited attention to their interplay. Integrated evidence remains scarce. Guided by the Person–Environment (P–E) Fit theory, the aim of this study is to conduct a narrative review of research on aging in older adults from a health perspective, systematically examining the effects of intrinsic capacity, environmental support, and their interaction on healthy aging, thereby providing guidance for further research.

## 2 Methods

### 2.1 Theoretical framework

In 2015, the World Health Organization (WHO) defined healthy aging in the “World report on aging and health” as “the procedure of establishing and maintaining the functional capacity necessary for well-being in later life.” Intrinsic capacity denotes the composite of an individual’s physical and psychological ability that reflects overall health status. It interacts dynamically with physical and social environment to enable functional activities, most commonly manifested as the ability to execute activities of daily living (ADL) and levels of social participation (Rantanen, 2025). Functional ability defines the health-related attributes that empower individuals to live and act in alignment with their values and preferences. It represents both the external manifestation of intrinsic capacity and a key reflection of an individual’s ability in engaging with their surroundings. Thus, healthy aging emphasizes not only individual characteristics but also the crucial role of environmental support in fostering health and wellness in later life.

**Figure 1:**
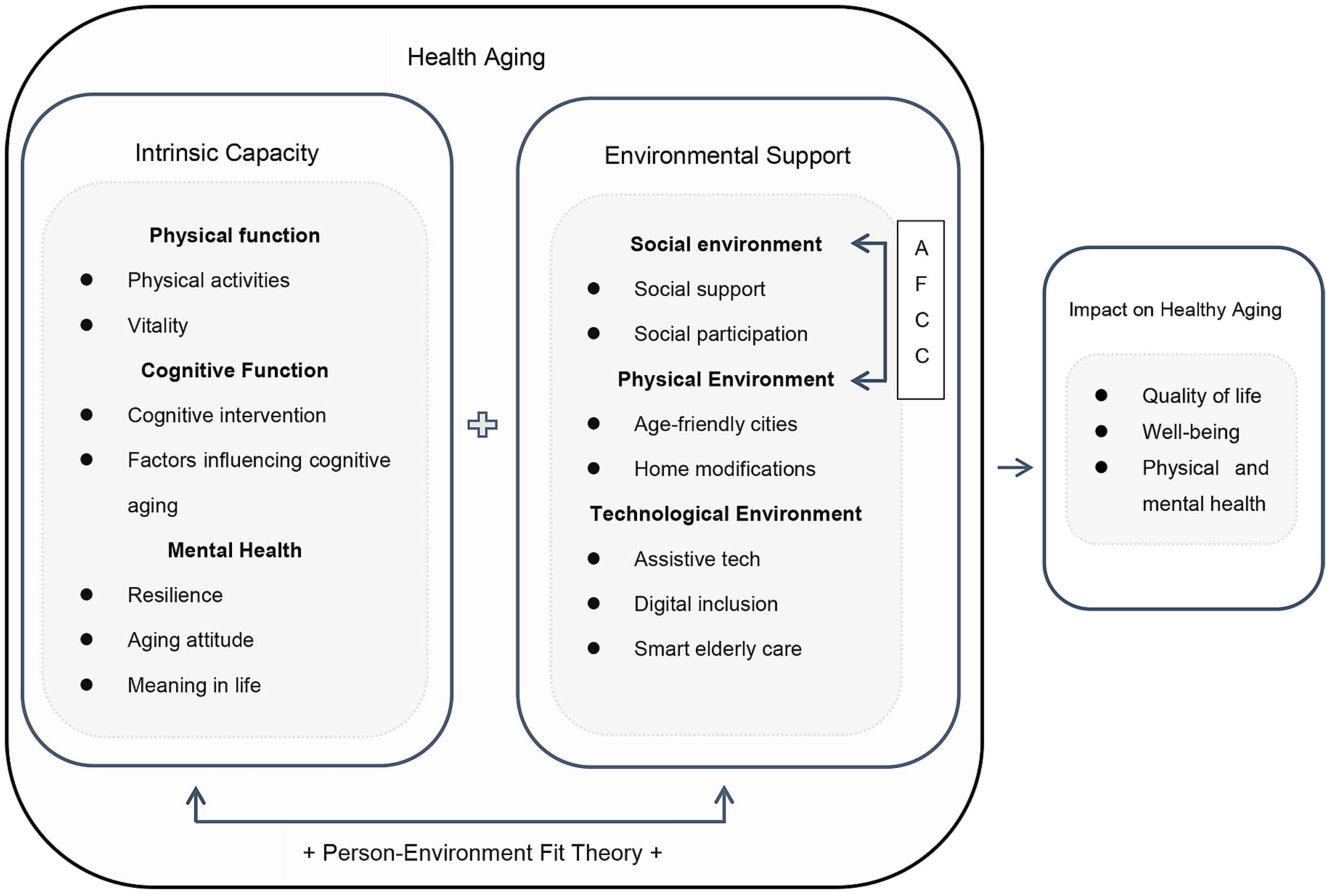
Research framework.

The Person–Environment Fit theory indicates that behavior and health outcomes are shaped not only by individual and environmental factors independently but also by their interaction (Kreiner, 2006). The combination of personal and environmental resources is considered a key determinant of health (Wahl et al., 2012). Modes of fit include compensatory and congruence. The compensatory form refers to the situation where, when intrinsic capacity is limited, environmental support is needed to achieve a satisfactory complementary level. The congruence form indicates that individual needs should be aligned with the provision of environmental resources (Carp and Carp, 1984). When there is a high degree of fit between the individual and the environment on certain characteristics, this relationship tends to yield positive outcomes (Merecz and Andysz, 2012). Conversely, when the balance between demands and capabilities exceeds an optimal range, negative emotions and maladaptive behaviors can arise. Therefore, this study constructs a theoretical framework based on the core concepts of healthy aging, combined with the Person-Environment Fit theory. It conducts a narrative review exploring the effects on healthy aging from three levels: intrinsic capacity, environmental support, and their interaction. The research framework of this study is shown in Figure 1.

### 2.2 Literature search strategy

A combination of subject headings and keywords was employed to search Chinese and English databases, including CNKI, Wanfang, PubMed, Web of Science, ScienceDirect, Sage Journals, APA PsycINFO, and ProQuest. The search period spanned from the inception of each database up to December 31, 2025. The search query included “intrinsic capacity,” “environmental support,” “healthy aging,” “older adults/the older people/the elderly/aged,” along with related concepts: terms related to dimensions of intrinsic capacity (e.g., physical function, cognitive function, mental health), terms related to dimensions of environmental support (e.g., social environment, physical environment, technological environment), and terms related to health, hedonic well-being (e.g., life satisfaction), and eudaimonic well-being (e.g., meaning in life).

### 2.3 Literature inclusion and exclusion criteria

Inclusion criteria: To be considered, studies had to be published in Chinese and English and report descriptions the effect of intrinsic capacity, environmental support, and their interaction on health and/ or on well-being. Additionally, further relevant studies were extracted by searching the reference lists of the identified articles. Given that this study aimed to perform a critical literature review rather than a systematic review, we iteratively updated the literature search strategy to ensure that the selected evidence was both rigorous and accurately addressed the core issue of how intrinsic capacity, environmental support, and their interaction influence the health of older adults.

Exclusion criteria: Unpublished papers (including conference papers, master’s theses, doctoral dissertations, commentaries, etc.); papers for which the full text could not be obtained.

### 2.4 Search results

After applying the inclusion and exclusion criteria, a total of 112 studies were included in the final analysis. Among these, 50 focused on intrinsic capacity, including physical function (*n* = 16), cognitive function (*n* = 19), and mental health and positive aging (*n* = 15). A further 59 studies examined environmental support, covering the social (*n* = 22), physical (*n* = 17), and technological environments (*n* = 17). In addition, 6 studies addressed the interaction between intrinsic capacity and environmental factors. Supplementary Table S1 provide a detailed summary of the main characteristics and key findings of the reviewed studies.

## 3 The mechanism of intrinsic capacity in healthy aging

Building on the International Classification of Functioning, Disability and Health framework, Cesari et al. (2018) identified five domains of intrinsic capacity based on existing evidence: motor, vitality, cognition, psychology, and sensory function. In view of this, an increasing number of studies have focused on how different dimensions of intrinsic capacity influence health outcomes in older adults attempting to elucidate the underlying mechanisms and provide empirical support for achieving healthy aging.

### 3.1 Physical function

Declines in physical function among older adults are characterized by reduced mobility, elevated risk of malnutrition, and heightened vulnerability to chronic illnesses. These changes can significantly compromise their abilities to live independently. This was demonstrated in several studies that regular physical activity not only helps prevent chronic illnesses but also lowers the risk of frailty and falls. Moreover, it can maintain key functional indicators like gait speed and grip strength, and improve body composition, including weight, waist circumference, and body mass index (Langhammer et al., 2018). Beyond its physical benefits, regular physical exercise can also elevate mood, boost self-esteem, and promote social interaction (Mahindru et al., 2023). Multicomponent physical exercise is widely recognized as an effective health-promoting approach. It typically integrates balance training, resistance exercise, and aerobic activity, incorporating gait, coordination, and overall functional training. Various leisure activities such as dancing, Tai Chi, gardening, or athletics can also be regarded as important forms of multicomponent exercise (Piercy et al., 2018). Relevant research indicates that such integrative forms of physical activity yield benefits across multiple health dimensions. For example, meta-analyses indicate that Tai Chi not only enhances cognitive function in older individuals but also improves quality of life and alleviates cancer-related symptoms (Wayne et al., 2014, 2018). Furthermore, a systematic review shows that resistance training and multimodal exercise interventions significantly improve physical function, while regular engagement in meditative movement practices contributes to better sleep quality (Di Lorito et al., 2021).

However, to gain a more accurate understanding of the relationship between physical activity and health outcomes, more precise and systematic monitoring of physical activity levels in older adults is needed. Physical activity level is commonly recorded in research using either objective monitoring devices or self-report questionnaires (Dowd et al., 2018). While devices such as accelerometers and pedometers collect objective data by directly tracking movement, self-report tools (e.g., questionnaires) allow for larger-scale data collection and are often more feasible than objective monitoring (Kurita et al., 2021). Current research still faces challenges regarding the suitability of tools for measuring physical activity levels among older adults. Most physical activity assessment methods were developed for younger or middle-aged populations, with relatively few instruments specifically designed for older individuals, such as the short form of the International Physical Activity Questionnaire. Although the validity of the IPAQ-SF has been illustrated by older adults, it may exaggerate the duration of physical activity across all intensity levels. Some subjective self-report methodologies (e.g., questionnaires, logs, and recalls) might be cognitively challenging for older adults, potentially resulting in biases in memory, response accuracy, and question interpretation, thereby reducing the validity of reported daily activity levels (Cabo et al., 2025).

The dimension of “vitality” also faces significant challenges during the aging process. Vitality is operationally defined as the physiological function related to energy metabolism and nutritional status. It can be served as a foundational driver supporting other domains of intrinsic capacity. Numerous studies have explored the potential benefits of nutritional status in promoting health, maintaining physical function, and preventing various detrimental health outcomes in older adults. Research indicates that favorable nutritional status is essential for supporting immune function (Alwarawrah et al., 2018), preserving musculoskeletal health, and preventing chronic diseases (Struijk et al., 2020; Rodríguez-Mañas et al., 2023). Instead, malnutrition has been identified as a key mediator of adverse outcomes, including functional decline (Villafañe et al., 2016), reduced quality of life (Marshall et al., 2014), and cognitive decline (Melzer et al., 2021). As there is currently no universally acknowledged definition of malnutrition, and it may vary over time and across physiological conditions, no single measurement tool is considered a definitive diagnostic standard (Skipper, 2012). Consequently, considerable heterogeneity exists in diagnostic criteria across studies, increasing the risk of misclassification and diagnostic error.

### 3.2 Cognitive function

As a key determinant of healthy aging, cognitive function can predict older adults’ self-perceived health (McHugh and Lawlor, 2016) and specific aspects of mental health, like lower levels of pessimism in later life (Taylor et al., 2017). Therefore, maintaining favorable cognitive function is essential for successful aging. Current research on cognition and healthy aging primarily focuses on two areas. On the one hand, studies have examined the determinants of cognitive aging from a multidimensional perspective, including lifestyle factors (Bloomberg et al., 2024; Jing et al., 2025), cognitive reserve (Iraniparast et al., 2022), and nutrition-related factors (e.g., dietary strategies, key nutrients, and dietary supplements) (Stavrinou et al., 2020); on the other hand, research has increasingly focused on interventions targeting cognitive decline and their effectiveness, including exercise interventions (Erickson et al., 2011), cognitive training (Li et al., 2025), and nutritional interventions (He et al., 2025).

Some evidence suggests that multiple lifestyles and behavioral factors like regular physical activity, non-smoking, limiting alcohol consumption, and maintaining frequent social engagement are associated with a reduced risk of cognitive decline and dementia (Bloomberg et al., 2024; Livingston et al., 2024).

The underlying mechanism may be because lifestyle-related protective behaviors can buffer the adverse effects of aging and disease-related brain changes by promoting the accumulation of cognitive reserve (Song et al., 2022). However, it is important to note that these behaviors are often interconnected and co-exist in clusters, forming behavioral clusters that jointly influence health outcomes (Spring et al., 2012). Currently, research exists showing that the relationship between combined lifestyle factors and cognitive function originates from Western countries (Gao et al., 2024), and the effects of risk factors for cognitive decline may vary across different countries and ethnic groups (McHugh et al., 2017; Mukadam et al., 2023). Thus, future research should adopt a more integrative perspective, examining the synergistic and cumulative effects of multiple lifestyle behaviors on cognitive function, rather than focusing solely on the independent contribution of single factors. In addition, more empirical studies are needed in the Chinese context to address existing research gaps and to better capture the influence of sociocultural differences on these associations.

How to effectively delay decline through cognitive interventions is also a key focus of current research. Common intervention approaches in existing studies mainly include cognitive training, nutritional interventions, and exercise interventions. Cognitive training, as the most direct approach, encompasses a range of techniques, including multi-target tracking and memory exercises, paper and pencil tasks (e.g., puzzles such as Sudoku or word games), as well as online and device-based training formats (Harvey et al., 2018). Several studies have demonstrated that computerized and traditional cognitive training can produce clinically meaningful improvements in specific cognitive domains among older adults (Kueider et al., 2012). However, Butler et al. (2018) point out that although improvements are observed in trained domains, these improvements do not necessarily transfer to overall cognitive function. This finding suggests that the mechanism of cognitive training may not directly enhance overall cognitive capacity but rather act indirectly by promoting an active lifestyle that maintains cognitive ability and efficiency. Consequently, the benefits of cognitive interventions may be more evident in domain-specific improvements and the accumulation of cognitive reserve, while their broader impact on overall cognition and the assessment tools require further investigation.

Concurrently, nutritional interventions have emerged as an important non-pharmacological approach due to their potential role in protecting cognitive function and delaying cognitive decline (Robinson, 2018). Nutritional interventions typically involve either specific dietary components (e.g., vitamin B, selenium, and omega-3 fatty acids) or synergistic dietary patterns rich in bioactive compounds (e.g., the Mediterranean diet). There are, however, several limitations in current research. For example, there is a lack of consensus on optimal nutritional combinations and an unclear understanding of the specific biological mechanisms that explain how nutritional interventions protect cognitive function. Despite observational study has identified associations between certain dietary patterns and a reduced risk of cognitive decline (Scarmeas et al., 2018). It further speculates that specific nutrients may play key regulatory roles, however, a lacking systematic examination of their underlying mechanisms and the interactions between nutritional components may lead to underestimation of their true effects. Thus, future research should aim for rigorous methodological approaches by integrating the combined effects of multiple nutrients and non-nutritional components to better elucidate their mechanisms and intervention pathways in cognitive aging.

### 3.3 Research on mental health and positive aging

Previous studies on mental health mainly adopted a pathological perspective, characterizing mental health as the lack of psychiatric diagnoses or symptoms and emphasizing non-pathological states (Vaillant, 2012). With the development of positive psychology, the research perspective progressively transitioned from a negative to a positive orientation, thus giving rise to the concept of positive psychological well-being. Against this backdrop, researchers have begun to focus on the protective role of positive psychological attributes (e.g., resilience, aging attitude, and meaning in life) in maintaining and promoting individual health. In addition, researchers further explore the application of positive psychological interventions in chronic disease management, thereby expanding both the theoretical scope and practical pathways of mental health research.

Resilience denotes an individual’s capacity to manage hardship in ways that protect health, wellness, and life satisfaction (Netuveli et al., 2008). It may serve as a key mechanism for mitigating the detrimental impacts of stressors on physical-mental health in later life (Laird et al., 2019). This was demonstrated in a few studies that resilience plays a critical role in the aging process (Resnick, 2014; MacLeod et al., 2016; Treichler et al., 2020). Resnick (2014) pointed out resilience is a dynamic process by which older adults bounce back from hardship and reintegrate and ideally evolve from the experience. As shown in the research focusing on the health outcomes associated with high resilience, despite facing various adversities, older adults exhibiting elevated levels of resilience tend to report better quality of life, improved mental health, and an increased likelihood of achieving subjectively perceived successful aging (Laird et al., 2019). Treichler et al. (2020) developed targeted psychosocial interventions aimed at strengthening resilience among older adults in coping with stressors and other negative impacts of natural aging. However, MacLeod et al. (2016) pointed out that existing research has not fully explained individual differences in how older adults cope with the unique adversities of later life (in terms of number, severity, and impact), and the underlying mechanism remains unclear. Accordingly, there is a need to develop more tailored resilience-based interventions targeted at different types of adversity.

With the advancement of positive psychology, aging attitude, as a distinctive cognitive element in the self-perception of the elderly, has gradually become a key variable in mental health research. According to stereotype embodiment theory (Levy, 2009), the way in which people perceive their own aging will affect their behaviors, cognition, and social functions. These effects can have long-term impacts for both physical and mental well-being. A study by Quinn et al. (2009) demonstrated that individuals with more favorable aging attitudes were more inclined to participate in health-promoting behaviors and reported higher levels of subjective well-being. In addition, compared to older adults with negative aging attitudes, those with positive attitudes exhibit faster walking speed, greater muscle strength (Deshayes et al., 2021) and are more likely to preserve a healthy lifestyle and stronger self-care ability. Existing research has largely drawn on investigating the direct correlation between attitudes toward aging and health outcome variables (Bryant et al., 2012; Nakamura et al., 2022). However, there remains a lack of research that systematically analyzes the internal structure and underlying mechanisms of attitudes toward aging from a multidimensional perspective. As a complex psychological construct, aging attitude typically encompasses three components: a cognitive dimension (beliefs and expectations), an affective dimension (emotional responses and evaluations), and a behavioral dimension (patterns of interaction and engagement). However, these dimensions are often not clearly distinguished in empirical research. The Attitudes to Aging Questionnaire (AAQ) by Laidlaw et al. (2007) is one of the most commonly used instruments for assessing attitudes toward aging. It is based on a three-factor model that evaluates attitude across three dimensions: psychosocial loss, physical change, and psychological growth. However, relatively few studies have conceptualized and measured aging attitudes from the perspective of their underlying attitudinal structure. Therefore, it is necessary to adopt an integrated cognitive–affective–behavioral framework to systematically examine the relationships between different dimensions of aging attitude and physical and mental health outcomes, thereby providing deeper insights into their underlying mechanisms and enriching the existing theoretical framework.

In reviewing the relationship between aging attitude and health outcomes, meaning in life, defined as another important positive psychological construct, represents a crucial protective role in promoting physical and mental wellness (Ness et al., 2014; Czekierda et al., 2017; Nie et al., 2023). Ness et al. (2014) conducted thorough narrative interviews and phenomenological analysis with 11 elderly women and 12 elderly men in rural central Norway. It revealed that despite being at a critical stage of life, these older individuals were able to draw on inner resources to maintain a positive outlook on physical decline, loneliness, and various life adversities, and to pursue new meaning in the challenges of aging. Evidence from longitudinal studies further supports this perspective, indicating that the sense of meaning in life may enhance mental health and diminish suicide risk in elderly individuals (Heisel and Flett, 2016). Although more and more studies emphasize the important role of purpose and meaning in facilitating optimal aging, empirical findings regarding the role of meaning in life remain inconsistent. A five-year longitudinal study demonstrated that meaning in life did not exert a significant protective effect against depression (Hedberg et al., 2010). This inconsistency might stem from individual differences in physiological, psychological states, as well as social environments (Nie et al., 2023). In this context, direct relationship between variables may be insufficient to fully explain the underlying mechanism. For example, one study pointed out that aging attitude might serve as an important mediator between meaning in life and mental health (Chen et al., 2022). However, the role of specific dimensions of aging attitude involved here (cognition, affection, and behavior) in promoting mental health through a sense of meaning in life is as yet unknown, opening avenues for future research.

## 4 The role of environmental support in healthy aging

The World Health Organization introduced the term of Age-Friendly Cities and Communities (AFCC), defined as communities where policies, services, and infrastructures related to physical and social environment are formulated to support and empower older people to “age actively”—that is, to live securely, maintain good health, and continue to engage fully in society (World Health Organization, 2007). This framework highlights the importance of both social environment (SE) and built environment (BE) and encompasses environments at both community and city levels. The social environment focuses on human-centered attributes, such as social support, healthcare services, respect and social inclusion, and social participation. In contrast, the built environment prioritizes physical elements, including housing, outdoor spaces, and infrastructure.

### 4.1 Social environment

#### 4.1.1 Social support

As a core element of the external environment, the concept of social support was initially introduced in the psychiatric literature. It is characterized as information that convinces the individual of being valued, cherished, and integrated into a network of reciprocal responsibilities (Cobb, 1976). With advances in this field, the classification of social support has diversified based on respective research needs. From a functional perspective, Flannery (1990) categorized social support into emotional support, instrumental support, informational support, and companionship. Cohen and Wills (1985) further distinguished these types by suggesting the first two categories as general resources for coping with common issues, whereas the latter two are more targeted resources. Social support has also been classified by the support subjects into formal and informal types (Mindel et al., 1986). Formal social support refers to assistance provided to vulnerable groups by governments, institutions, communities, and other formal organizations, such as pension systems and healthcare services (Chi and Han, 2022). Informal social support includes emotional, behavioral, and informational support from family members, friends, neighbors, and colleagues (Tao and Shen, 2014). A substantial body of research, both domestically and internationally, has examined the correlation between social support (formal vs. informal) and health outcomes. Results indicated the positive effects of social support on health, particularly mental health.

In terms of informal social support, scholars have examined the implications of non-institutionalized support from community, family, and social organizations on the health of older adults. With the shrinking size of families and the increasing prevalence of nuclear family structures, the role of family support has gradually weakened (Cheng, 2016). An “age-friendly” community can promote psychological well-being by providing infrastructure that supports meaningful social connections (Nieboer and Cramm, 2018). Sun et al. (2016) found that access to community healthcare services positively contributes to mental health, whereas insufficient public services may undermine these effects. Moreover, life course theory suggests that older adults derive greater benefits from support obtained from spouses and close family members. This is probably because that compared with younger individuals, who tend to perceive time as expansive, older adults are more aware of the limited time remaining and therefore prioritize emotionally meaningful relationships (Carstensen, 1998). However, empirical findings are not entirely consistent. Some studies indicate that support from friends may have a stronger effect on well-being in later life (Huxhold et al., 2014). As family support may decline due to the loss of spouses or close relatives, older adults tend to rely more on friendships as a primary source of support. Given these contradictory findings, further empirical research is needed to clarify how different types of social support influence health outcomes across individuals.

Regarding formal social support, researchers have focused on the effects of social security systems such as pension and health insurance on the well-being of older adults. Internationally, many countries have implemented comprehensive aging policies. For example, Japan formally implemented the “Long-Term Care Insurance system” in 2000, integrating medical and care services to enhance health security for older populations (Iwagami and Tamiya, 2019). Nordic countries have constructed community-based care systems to achieve a seamless connection between aging in place and health promotion through coordinated services (Agerholm et al., 2023). Domestic scholars have found that the social security system has a positive impact on both physical and mental health. Endowment insurance improves health outcomes by enhancing their capacity and levels in areas like access to food, healthcare expenditure capacity, and overall well-being through pension payments (Liu and Wang, 2017). Medical insurance has also been shown to improve mental health by promoting physical health, enhancing security expectations, and increasing life satisfaction (Li et al., 2022). Zhou et al. (2018) observed that the existing social security system in China is conducive to fostering the mental well-being of rural inhabitants, but the effects vary considerably across different types of social insurance. Pension insurance presents a significant beneficial effect on mental health. Although no significant difference is observed between individuals who contribute to pension insurance and those without such coverage, recipients of pensions under the New Rural Pension Scheme exhibit significantly better mental health compared to those without pension insurance. The medical insurance system also appears to have a beneficial effect on mental health, its impact does not reach statistical significance, and the coefficient for the New Rural Cooperative Medical Scheme is basically nonsignificant. Overall, existing research has not reached a consensus on the impact of China’s current social insurance system on mental health. Again, more empirical evidence is needed to reliably answer these, as yet, the differential effects of different institutional types.

#### 4.1.2 Social participation

The predictive role of social participation in health conditions has become a prominent research focus in aging research (Fain et al., 2022). There is no universal consensus on the definition of social participation. Internationally, it is often conceptualized from perspectives such as resource exchange, interpersonal interaction, and engagement in activities (Dehi and Mohammadi, 2020). Levasseur et al. (2010) have systematically classified social participation and emphasized that it includes not only daily interaction with others but also social, economic, cultural, and political activities. The core of social participation is to establish continuous connections and interaction between older adults and society. Based on this framework, Wang (2011) further proposed that social participation can be categorized into four types: daily life participation, volunteer/public service participation, economic participation, and political participation. These categories, respectively, reflect the extent of older adults’ involvement in family life, community affairs, employment, and civic engagement.

Social participation has a positive impact on the physical and mental well-being of older adults. It provides opportunities to expand social networks and enhance emotional exchange, enabling individuals to obtain greater emotional support and experience lower levels of loneliness in later life and thus effectively alleviate depressive symptoms (Fu et al., 2017). Moreover, engagement in a diverse range of activities appears to have a stronger protective effect against depression than participation in a single type of activity (Sugihara et al., 2008). Some scholars have noted that different forms of social participation may exert distinct effects on mental health. A survey of middle-aged and elderly people aged 45 and above found that voluntary social participation has less impact on the maintenance of daily functioning and the reduction of depressive symptoms than participation in recreational or cultural activities (Du, 2022). However, excessively frequent or passive participation may lead to negative emotional outcomes (Fang et al., 2020). Self-recreational leisure activities can help older adults maintain cognitive function, physical function, and psychological well-being, thereby contributing to successful aging (Sala et al., 2019). Notably, the effects of work-related social participation are closely linked to individuals’ motivations for participation. Older adults who engage in labor passively to supplement income or support family members are more likely to report higher levels of negative feelings (Yu et al., 2016). Conversely, those who participate in work voluntarily for self-fulfillment are more beneficial to mental health through both increased income and a sense of personal value (Zhou et al., 2023).

A considerable body of literature has documented the correlation between social participation and mental well-being in older adults, yet there remain entails for further exploration. First, while the advantageous impacts of social participation on mental health are widely acknowledged, the outcomes vary depending on the type of participation, and not all forms yield positive outcomes. This highlights the need for more detailed classifications and analyses of participation types. Additionally, existing research rarely focuses on the impact of the quantity and frequency of social activities. Future research should therefore adopt longitudinal designs to better explore how various types and levels of social participation influence health outcomes over time.

### 4.2 Physical environment

The core of age-friendly cities lies in fostering and maintaining functional ability through supportive environments, promoting individual well-being in later life, and providing the indispensable support for healthy aging (Alley et al., 2007; World Health Organization, 2007; Torku et al., 2021). In recent years, initiatives to develop age-friendly cities and communities have gained increasing attention (Buffel et al., 2012). In the United States, Age-Friendly Communities (AFCs) encompass domains such as housing planning, health and well-being, accessible services, and community engagement (Scharlach, 2012); the United Kingdom advocates the idea of “Lifetime Neighborhoods,” encompassing housing, free transportation, green spaces, and community safety (Rooney et al., 2013). In China, the State Council’s *National Medium- and Long-Term Plan for Actively Addressing Population Aging* highlights the development of age-friendly cities, including: (1) improving the suitability of residential environments for older adults by incorporating accessibility and age-appropriate renovations into urban renewal and the renovation of old urban residential communities; (2) facilitating older adults’ daily mobility by strengthening barrier-free modifications in public and residential facilities, such as parks, community service centers, rest areas, and information infrastructure. Within the dynamic interaction between physical and social environments, the physical environment serves as a basis for social interaction and plays a vital role in shaping well-being in later life (Wang et al., 2023). Therefore, further examination of how various elements of the physical environment contribute to improving older adults’ health and well-being is essential. Furthermore, additional empirical investigations are required to address existing research gaps and to better capture the influence of sociocultural differences on these associations.

Empirical evidence suggests that living in a supportive environment characterized by a higher degree of age-friendly features and better person–environment fit is associated with better health outcomes (Choi, 2020). For example, community open spaces such as parks can enhance the emotions of elderly people and reduce loneliness. Green spaces and recreational areas provide older adults with more opportunities to socialize with friends and neighbors. This can also develop the interpersonal relationships of older adults and cultivate a sense of emotional connection and belonging (Tang et al., 2025). In addition, green spaces not only serve as ideal venues for social engagement and outdoor activities but can also directly promote physical health and improve life satisfaction when equipped with age-friendly exercise and recreational facilities (Pan et al., 2024; Tang et al., 2025). Several other factors promoting the health of older adults have been identified, including gait accessibility, esthetics, safety, opportunities for leisure, and lower levels of noise (Gao et al., 2016; Cerletti et al., 2021). Transportation and mobility are also key points in discussions of the physical environment, particularly for older adults who cannot drive (Fitzgerald and Caro, 2014). Convenient and accessible public transport facilities have been found to reduce mobility barriers for older adults, enabling them to participate in more social activities (Zhang and Yang, 2024).

At present, aging in place remains the preferred option for older individuals. However, the atmosphere they live in often presents safety hazards and lack adequate assistive features, which not only increases the risk of falls but also hinders the performance of daily activities. Consequently, when the living environment places excessive demands on individuals with reduced functional abilities, disability and dependency increase, necessitating home modifications tailored to their specific needs (Byrnes et al., 2006). Research indicates that age-friendly home modifications, like installing handrails, adding non-slip flooring, and removing steps, can substantially decrease the likelihood of accidents, particularly falls (Wiseman et al., 2021) and alleviate the effects of physical decline (Petersson et al., 2008). Secondly, age-appropriate housing modifications can contribute to the well-being of older adults by fostering positive aging attitudes. Studies indicate that age-friendly housing modifications provide supportive conditions to strengthen independent living, mitigate the negative effects of age-related stereotypes, and encourage more positive responses to the aging process (Che et al., 2024; Lyu et al., 2025). However, as far as we are aware, existing research on home modifications for older adults has primarily focused on fall prevention and the maintenance of physical function. There is still some uncertainty regarding the assessments of their overall effects for fostering the well-being of older adults. However, in a super-aged society, a more comprehensive strategy that extends beyond physical safeguards to systematically incorporate functional independence, quality of life, caregiving burden, cost-effectiveness, and social participation is required in further research.

### 4.3 Technological environment

Over time, technology has increasingly become an essential component of contemporary and future societies. However, the World Health Organization’s eight-domain framework for age-friendly cities does not explicitly incorporate technology. To address this gap, Van Hoof and Marston (2021) highlighted the absence of a technological dimension in the WHO framework and proposed insights and recommendations for its extension. The state council of China also mentions in policy documents that building an age-friendly society requires the development of an inclusive digital society and the establishment of digital support systems as well as assistive technology environments. This will help older adults integrate more effectively into the digital age.

Assistive technology is an innovative concept integrating technology into residences to maintain or even enhance residents’ functional health, safety, and quality of life. Khosravi and Ghapanchi (2016) argued that existing research on the role of assistive technologies in supporting adults aged 60 and above has primarily focused on issues such as chronic diseases, dementia, social isolation, depression, poor medication management, and overall poor health status. Technologies designed to address these challenges include general information and communication technologies (ICTs), robotics, mobility aids, telemedicine, sensor technologies, and medication management applications that improve treatment accuracy. Empirical evidence indicates that mobility aids can significantly improve movement among individuals with physical impairments. Devices such as canes, walkers, manual wheelchairs, and powered mobility scooters are particularly beneficial for those who can walk intermittently but are primarily limited by endurance rather than balance or coordination (Sehgal et al., 2021). In addition, on the premise of maintaining physical-mental health and overall quality of life, emerging technologies like the Internet of Things (IoT), assistive robotics, artificial intelligence (AI), and health monitoring systems can support older adults in choosing their preferred living environments according to their own preferences. Ultimately, enabling both ambient assisted living and active assisted living (Calvaresi et al., 2017; Mostaghel and Oghazi, 2017).

Although many existing AT applications have traditionally targeted older adults with specific diseases or disabilities, their scope has expanded to support independent living at home, commonly referred to as aging in place, and recent reviews have specifically addressed this topic (Bergschöld et al., 2024). Nevertheless, some studies raise critical concerns, suggesting that while ATs offer substantial benefits, they may also entail potential drawbacks. These concerns include the possibility that technologies might undermine rather than enhance autonomy, as well as the limitations of AI systems trained on common characteristics of the majority, which may face difficulties in addressing diversity (O’Brolcháin, 2018). Additionally, some research has not adequately considered alternatives to aging in place, such as assisted living facilities, multigenerational living arrangements, or long-term nursing institutions. It should be noted that these are also worthwhile options for some individuals (Vasara, 2015; Fernández-Carro, 2016).

Simultaneously, how to reduce the digital divide and mitigate social exclusion through the development of digitally inclusive social environments has increasingly attracted scholarly attention. Digital inclusion extends and refines traditional digital divide theory by emphasizing the deep integration of digital technologies into everyday life and service provision, thus enabling seamless interaction between technology and daily living (Fisk et al., 2023). It can facilitate individuals’ participation in health management and decision-making to improve older adults’ quality of life (French and Richardson, 2017). Studies conducted both domestically and internationally have reached similar conclusions. These findings demonstrate that ICTs can enhance older adults’ quality of life across multiple aspects, including cognitive functioning, social integration, and community satisfaction (Ordonez et al., 2011; Wong et al., 2014). Winstead et al. (2013) observed that these benefits appear to be closely linked to the establishment of support networks through the internet. Studies have shown that the internet facilitates interaction between older adults and their family members, relatives, and friends, alleviates feelings of loneliness, and fosters self-efficacy (Wong et al., 2014). At the same time, it plays a crucial role in strengthening social connectedness by bridging older adults’ relatively isolated living contexts with their family members, including grandchildren, exerting a positive influence on their quality of life (Bobillier Chaumon et al., 2014).

In addition, smart elderly care has emerged as a key direction in the innovation of care services, with considerable potential to enhance quality of life and promote healthy aging. However, due to the notable deficiencies in older adults’ ability to access and utilize digital technologies, the actual utilization of such services remains constrained. Consequently, enhancing the accessibility and usability to digital technologies for older adults to construct an inclusive digital social environment is obviously important. The integration of digital technologies into eldercare services has been found to be more likely to reduce social care expenditures and improve the quality of life and mental health of older adults (Golant, 2017). Existing studies have largely focused on the role of technological characteristics and environmental factors in shaping technology adoption intentions (Pal et al., 2019). While digital technologies can assist older adults in improving health management and quality of life, existing studies have primarily concentrated on the role of technological features and environmental factors in shaping technology adoption among older adults (Pal et al., 2019). The mechanisms through which individual-level factors such as digital literacy and attitude toward aging influence technology acceptance and use remain insufficiently explored.

## 5 The interaction mechanism between intrinsic capacity and environment in healthy aging

### 5.1 Theoretical explanation: how does the interaction between intrinsic capacity and environment affect healthy aging?

Currently, there remains limited empirical evidence that links the interaction between intrinsic capacity and environmental support to the functional capacity of older adults. This section applies the Person-Environment Fit theory and environmental compensation mechanism to explain how intrinsic capacity and environmental factors interact to affect healthy aging.

#### 5.1.1 Person-environment fit theory

Previous research on Person–Environment (P–E) fit commonly utilizes Lawton’s ecological model of aging. This theory asserts that older adults’ behaviors are determined by the interaction between intrinsic capacities and environmental resources. However, Kahana (1975) argued that the ecological model neglects individual needs. Consequently, they proposed a congruence model. This model emphasizes that the alignment between intrinsic capacity and environmental resources is crucial to maintaining adaptive behaviors, psychological health, and well-being. A study by Choi (2020) supported this view. This study explored the importance of age-friendly community features and person-environment fit for the health of older adults. By using a multidimensional approach aligned with the World Health Organization’s eight environmental domains, the study provided comprehensive evidence that higher perceived accessibility of age-friendly facilities and better person–environment alignment in the “outdoor spaces and buildings” domain were significantly associated with higher self-rated health and reduced risk of functional limitations. This finding aligns with the previously reviewed literature on the impact of environmental support on health (Petersson et al., 2008; Wiseman et al., 2021; Tang et al., 2025).

The Person-Environment-Occupation (PEO) proposed by Law et al. (1996) further emphasizes the dynamic interaction between individuals, environment, and occupational engagement. Fang et al. (2023) applied this model to identify potential pathways linking personal and environmental factors to activities of daily living performance. This finding indicated that physical environments (e.g., handrails) and social environments (e.g., family support) mediated the relationship between personal factors (e.g., functional impairments) and ADL performance. Hewston et al. (2021) similarly employed the PEO model to describe how frailty (personal) and living environments (environmental) influence occupational performance, thereby improving frailty-related activity capacity. It found that mobility aids and other assistive devices effectively mitigated activity limitations caused by physical decline, thus enhancing both mobility and daily activity performance within living spaces. Results demonstrated that environmental factors could compensate for intrinsic capacity deficiencies by providing external support resources, thereby improving functional ability. Regarding the technological environment, assistive devices mediate the relationship between mobility limitations and ADL performance. Thus, this can be understood from the perspective of person–environment fit and corroborates findings from the previous literature review.

#### 5.1.2 Environmental compensation mechanism

The Person-Environment Fit theory typically categorizes person-environment fit into two forms: compensatory and congruent. The compensatory form refers to the use of environmental supports to achieve an optimal complement when personal capacities are limited, whereas the congruent form reflects the alignment between intrinsic capacity and environmental resources (Carp and Carp, 1984). Within this framework, environmental factors can act as buffers, mitigating the negative effects of cumulative disadvantages or functional decline on health and well-being, and can serve as moderators to compensate for individual deficits (Fang et al., 2023), thereby attenuating the long-term impact of adverse factors on health outcomes. Research has demonstrated that especially for individuals facing cumulative socioeconomic disadvantage and declining social status, community infrastructure plays a critical role in slowing frailty progression. A study by Liu et al. (2023) verified this conclusion. Based on life-course and person–environment fit perspectives, this study investigated how community resources moderated the relationship between life-course socioeconomic disparities and frailty trajectories over a seven-year follow-up. This finding indicated that community environmental resources (basic infrastructure and charitable organizations) exerted a protective effect against the progression of frailty and mitigated the adverse impact of cumulative SES vulnerability experiences across the lifespan.

In addition, the residential environment is also a critical moderator in the process of preserving the health of older adults. Fang et al. (2023) observed that physical home environments (e.g., accessibility features and handrails) and family support can moderate the relationship between mobility impairments and ADL performance. Building on this, Park et al. (2017) further validated the critical role of the environment from a person–environment fit perspective. This research examined three core dimensions of aging-in-place including individual factors (e.g., income), environmental factors (e.g., housing), and adaptation (e.g., self-rated health) to explore how the fit between poverty status and age-friendly housing affects health outcomes. It implied that for low-income older adults living alone, residing in age-appropriate housing or age-friendly communities could, to some extent, compensate for their limited socioeconomic resources and significantly improve self-rated health and quality of life. In other words, even when intrinsic capacities decline or resources are insufficient, a well-configured environment can functionally substitute for these deficits by providing service support, opportunities for social interaction, and safe, accessible physical spaces, thereby promoting adaptive aging. Park and Lee (2017) further confirmed the moderating and compensatory roles of supportive physical and social environments. This may compensate for or partially offset the early-life disadvantageous situations and mitigate the long-term impact of disadvantageous circumstances on health and well-being.

## 6 Conclusion

This paper primarily focuses on the two core dimensions proposed by healthy aging, particularly the dynamic interaction between intrinsic capacity and environmental support within the person–environment fit framework. Prior studies frequently treated intrinsic capacity and environmental support as independent constructs or emphasized their separate roles in aging, thereby somewhat neglecting their interaction. Drawing on a health perspective, this review synthesizes the existing literature to integrate these two constructs and elucidate how environmental support moderates the relationship between intrinsic capacity and functional ability, ultimately promoting older adults’ health and well-being. Although a growing body of research focuses on the interactive effects between intrinsic capacity and environmental support, several limitations remain that require further investigation.

From a P–E fit perspective, intrinsic capacity primarily refers to individual characteristics, encompassing biological health, sensory capacities, and physical competence (Lawton, 1982), with a particular focus on attributes specific to the elderly. With advancing age, declines in these capacities increase dependence on environmental support. Simultaneously, structural factors such as socioeconomic status exhibit cumulative effects over an individual’s life course, and this long-term accumulation of disadvantage can further exacerbate individual differences in health outcomes. Existing P-E fit research predominantly focuses on health-related individual attributes. However, additional aspects of individual resources and limitations in older adults, such as socioeconomic status or other social stratification variables, are equally valid theoretical constructs in person-environment fit research. These factors not only influence the availability of individual resources but also significantly shape the degree of fit between the intrinsic capacity and environment, and thus these should be considered key analytic dimensions in future person-environment fit research. Moreover, the foundation of the P–E fit approach lies in the dynamic relationship between personal capacity and the environment. Future research could employ longitudinal study designs for monitoring changes in P–E fit over the life course, thereby more accurately determining whether alterations in person–environment alignment drive, or are driven by, health outcomes.

Regarding intrinsic capacity, the dimensions are not independent constructs but are interrelated and operate synergistically, jointly shaping recovery trajectories and long-term health outcomes. Existing evidence suggests that tailored multidomain interventions like physical exercise and nutritional optimization can bolster resilience, thereby improving clinical outcomes. Meanwhile, psychological and social factors also play an important role in strengthening intrinsic capacity. For example, strategies such as stress management, social support, and cognitive training can further enhance resilience, and these psychological interventions may act synergistically with improvements in physical and biological markers (Grigoraș et al., 2025). However, systematic investigations of multidimensional, integrated, and personalized intervention strategies remain scarce. In particular, there is a lack of in-depth analysis of the dynamic interrelationships among intrinsic capacity dimensions and the mechanisms through which they influence intervention effectiveness. Therefore, future research should develop more targeted and integrated intervention strategies and measurement tools suitable for the elderly in China.

With regard to environmental factors, early studies primarily focused on a single dimension of the physical environment. Recently, however, research has increasingly expanded to include social and technological environments, leading to a more comprehensive and multidimensional understanding of environmental characteristics. Existing studies commonly draw on the framework of age-friendly communities and cities proposed by the World Health Organization, emphasizing the integrated development of physical, social environments and their combined impact on the well-being of older adults. Meanwhile, with the acceleration of digitalization, the technological environment has emerged as an essential issue that affects older individuals’ quality of life and social integration. The development of age-friendly communities should therefore extend beyond optimizing physical and social environments to include the construction of digital environments, enabling older adults to better engage with the digital society. However, this dimension is not explicitly addressed in the WHO’s eight-domain framework for age-friendly cities (Van Hoof and Marston, 2021). Therefore, in advancing age-friendly community development, it is essential to adopt a holistic perspective that considers the interplay among physical, social, and technological environments.
