# Supplementary material for: The current status and implications of aging research from a health perspective
Source: Front Psychol. 2026 May 21;17:1775885. doi: 10.3389/fpsyg.2026.1775885 (PMC13233426; doi:10.3389/fpsyg.2026.1775885)
Supplement: Supplementary file 1 [file Table_1.DOCX]

**Supplementary Table 1:** Summary of the characteristics and key findings of the included studies

| **No.** | **Author** | **Publication years** | Country | **Research type** | **Key focus** | **Main findings** |
| --- | --- | --- | --- | --- | --- | --- |
| 1 | Wayne et al.  (2014) | 2014 | USA | Systematic review and meta-Analysis | Physical activities (Tai Chi) | Tai Chi may offer a safe, non-pharmacological approach to enhancing cognitive function in older adults. |
| 2 | Wayne et al.  (2018) | 2018 | USA | Systematic review and meta-Analysis | Physical activities (Tai Chi and Qigong) | Tai Chi and Qigong shows promise in addressing cancer-related symptoms and Quality of life of cancer survivors. |
| 3 | Piercy et al.  (2018) | 2018 | USA | Special Communication | Physical activity (PA) | The Physical Activity Guidelines for Americans, 2nd edition, provides information and guidance on the types and amounts of physical activity that provide substantial health benefits. |
| 4 | Langhammer et al.  (2018) | 2018 | Norway | Editorial | Physical activity (PA) | PA and exercise play an important role in the primary, secondary, and tertiary prevention, in the management of diseases, to counteract sarcopenia and falls as well as improving physical performance and activities of daily living. |
| 5 | Dowd et al.  (2018) | 2018 | Europe | Systematic review | Physical activities (Techniques for physical activity measurement) | Objective measures of physical activities demonstrate less variability in properties of methodological effectiveness than self-report measures. |
| 6 | Di Lorito et al.  (2021) | 2021 | UK | Systematic review (Meta-analysis) | Physical activities (Exercise interventions) | Exercise interventions for older adults are extremely diverse, which seem to suggest that the most effective interventions were resistance training, meditative movement interventions, and exercise-based active videogames. |
| 7 | Kurita et al.  (2021) | 2021 | Japan | Community-based cohort study | Physical activities | The Physical Activity Questionnaire Short Form can be an option to PA assessment tools such as accelerometers that objectively measure PA to roughly predict the risk of disability caused by physical inactivity. |
| 8 | Mahindru et al.  (2023) | 2023 | India | Review | Physical activity (PA) | The effects of exercise on mental health have been shown to be beneficial. Physical exercise improves depressive and anxiety symptoms. |
| 9 | Cabo et al.  (2025) | 2025 | Portugal | Parallel-group, randomized controlled trial | Physical activities (Physical exercise) | Several outcomes—such as physical activity levels, enjoyment, and quality of life—relied exclusively on self-reported instruments, which may be influenced by recall bias, social desirability, or cognitive limitations. The absence of screening for literacy or cognitive status means that some participants may have had difficulty understanding or accurately completing the questionnaires. |
| 10 | Skipper  (2012) | 2012 | USA | Review | Vitality (Malnutrition) | This finding provides clinicians with definitions and clinical characteristics of malnutrition that can be used to quickly and consistently document malnutrition. The consensus statement provides researchers with a uniform set of criteria that can serve as the basis for epidemiologic and intervention studies |
| 11 | Marshall et al.  (2014) | 2014 | Australia | Systematic review | Vitality (Malnutrition) | Malnutrition in older adults admitted for rehabilitation has a negative effect on functional recovery and quality of life following discharge to the community. |
| 12 | Villafañe et al.  (2016) | 2016 | Italy | Cross-sectional study | Vitality (Malnutrition) | Nutritional status is associated with functional status in a cohort of elderly inpatients in a rehabilitative center. Both nutritional and functional status worsen with age. |
| 13 | Alwarawrah et al. (2018) | 2018 | USA | Review | Vitality (Nutritional Status) | Changes in nutritional status have a wide range of effects on the body, which can influence organ size, hormone, and cytokine levels, and immune cell populations and function. |
| 14 | Struijk et al.  (2020) | 2020 | USA | Prospective longitudinal cohort study | Vitality (Diet quality and risk of frailty) | The findings extend the well-known benefits of healthy diets to include the retardation of functional decline, and suggest that old adults adhering to such diets can prevent frailty and its subsequent adverse health outcomes. |
| 15 | Melzer et al.  (2021) | 2021 | Brazil | Review | Vitality (Malnutrition) | Micro-nutrients such as vitamins and trace minerals are also crucial for maintaining brain health. In contrast, deficiency or disturbances in any of these can be associated with brain dysfunction and contribute, at least in part, to the pathophysiology of several neurological disorders. |
| 16 | Rodríguez-Mañas etal.  (2023) | 2023 | USA | Perspective article | Vitality (Malnutrition) | A lifestyle of healthy nutrition and physical activity in early adulthood and midlife is key to aging well. It further supports healthy aging, preserves musculoskeletal function, and helps lessen effects of decreased energy intake with aging. |
| 17 | McHugh and Lawlor  (2016) | 2016 | Ireland | Empirical research | Cognitive function | Older adults' self-rated health may be related to their executive functioning and to their loneliness. Self-rated health appeared to improve over time, and the extent of this improvement was also related to executive functioning at baseline. |
| 18 | Taylor et al.  (2017) | 2017 | Ireland | Longitudinal study | Cognitive function | The findings highlight an important aspect of social functioning in the older population, and suggest that the discrepancy between objective isolation and felt loneliness may be associated with undesirable health outcomes such as cognitive dysfunction. |
| 19 | Spring et al.  (2012) | 2012 | USA | Randomized Controlled Trial | Factors influencing cognitive aging (Lifestyle) | Remote coaching supported by mobile technology and financial incentives holds promise to improve diet and activity. Targeting fruits/vegetables and sedentary leisure together maximizes overall adoption and maintenance of multiple healthy behavior changes. |
| 20 | Mukadam et al.  (2023) | 2023 | UK | Purely observational research | Factors influencing cognitive aging (Lifestyle) | Minority ethnic status confers a greater risk of dementia for many risk factors compared to White people. Hypertension, obesity, diabetes and low HDL seemed particularly important in South Asians and hypertension relatively more important in Black people. |
| 21 | Bloomberg et al.  (2024) | 2024 | UK | Longitudinal Study | Factors influencing cognitive aging (Lifestyle) | Differences in cognitive decline between lifestyles were primarily dependent on smoking status. |
| 22 | Livingston et al.  (2024) | 2024 | UK | Meta-analysis | Factors influencing cognitive aging (Lifestyle) | Modifying 14 risk factors (ie, vision loss and high cholesterol, less education, head injury, physical inactivity, smoking, excessive alcohol consumption, hypertension, obesity, diabetes, hearing loss, depression, infrequent social contact, and air pollution) might prevent or delay nearly half of dementia cases. |
| 23 | Gao et al.  (2024) | 2024 | China | Systematic review and meta-Analysis | Factors influencing cognitive aging (Lifestyle) | Adherence to a healthy lifestyle was associated with a lower risk of incident dementia and other cognitive outcomes. It is important to find a subtle balance between the benefits and adherence. |
| 24 | Jing et al.  (2025) | 2025 | China | Longitudinal study | Factors influencing cognitive aging (Lifestyle) | A greater number of healthy lifestyle behaviors (such as moderate alcohol consumption, nonsmoking, a healthy diet, and moderate sleep duration)was linked to better cognitive health. |
| 25 | Iraniparast et al.  (2022) | 2022 | USA | Longitudinal study | Factors influencing cognitive aging (Cognitive Reserve) | Those with higher levels of cognitive reserve indicators (educational attainment, academic performance, and written language skills) had a significantly greater chance of reversion from MCI to NC than progression from MCI to dementia. |
| 26 | Song et al.  (2022) | 2022 | USA | Systematic review | Factors influencing cognitive aging (Cognitive Reserve) | Lifestyle activity factors (physical and cognitive leisure activities) may contribute to CR and attenuate the damaging impact of brain changes on cognition. |
| 27 | S. Stavrinou et al. (2020) | 2020 | Cyprus | Randomized controlled study | Factors influencing cognitive aging (Nutrition-related factors) | A high-dose of specific omega-3 and omega-6 fatty acids supplementation, in combination with specific antioxidant vitamins, can be a potential nutritional modality for the prevention or possibly treatment of cognitive impairment and functional decline; thereby improving independence and quality of life for older individuals with MCI. |
| 28 | Erickson et al.  (2011) | 2011 | USA | Randomized controlled trial | Cognitive intervention (Exercise interventions) | Aerobic exercise training increases the size of the anterior hippocampus, leading to improvements in spatial memory. |
| 29 | Kueider et al.  (2012) | 2012 | USA | Systematic review | Cognitive intervention (Cognitive training) | Findings are comparable or better than those from reviews of more traditional, paper-and-pencil cognitive training approaches suggesting that computerized training is an effective, less labor intensive alternative. |
| 30 | Harvey et al.  (2018) | 2018 | USA | Review | Cognitive intervention (Cognitive training) | Computerized cognitive training has been shown in most studies to improve cognitive performance on untrained tests in healthy older people and in people with schizophrenia. |
| 31 | Butler et al.  (2018) | 2018 | USA | Systematic Review | Cognitive intervention (Cognitive training) | In older adults with normal cognition, training improves cognitive performance in the domain trained. Evidence regarding prevention or delay of cognitive decline or dementia is insufficient. |
| 32 | Li et al.  (2025) | 2025 | China | Meta-analysis | Cognitive intervention (Cognitive training) | Cognitive training supports cognitive improvements in older adults through increased activation of task-relevant and compensatory brain regions. |
| 33 | Robinson  (2018) | 2018 | UK | Review | Cognitive intervention (Nutritional interventions) | There is a need for wider recognition of malnutrition risk among older adults, including implementation of routine screening of nutritional status and early diagnosis. |
| 34 | Scarmeas et al.  (2018) | 2018 | USA | Review | Cognitive intervention (Nutritional interventions) | Certain nutrients or food ingredients, such as some B vitamins (particularly folate), flavonoids, vitamin D, and n-3 fatty acids, have the potential to benefit cognitive function. |
| 35 | He et al.  (2025) | 2025 | China | Meta-analysis | Cognitive intervention (Nutritional interventions) | Among patients with cognitive impairment, such as mild cognitive impairment and Alzheimer's disease, multi-ingredient nutrition may be the most effective approach to slow cognitive decline. |
| 36 | Netuveli et al., 2008 | 2008 | UK | Annual interview survey | Mental Health (Resilience) | Resilience is relatively rare and favors older women. It is fostered by high levels of social support existing before exposure to adversity. |
| 37 | Resnick  (2014) | 2014 | USA | Review | Mental Health (Resilience) | Resilience is not static, and interventions can be implemented to help older adults strengthen their resilience and thereby improve outcomes following challenging events. |
| 38 | MacLeod et al.  (2016) | 2016 | USA | Review | Mental Health (Resilience) | Resilience is most often viewed as a process rather than a personality trait; as such many older adults are capable of improving their resilience later in life. |
| 39 | Laird et al.  (2019) | 2019 | USA | Review | Mental Health (Resilience) | Research has elucidated both trait-like psychological factors that identify those at greatest risk and psychosocial processes that may be fruitful targets for intervention. |
| 40 | Treichler et al.  (2020) | 2020 | USA | Pragmatic trial | Mental Health (Resilience) | This study presented a novel psychological intervention, using pragmatic trial design, intended to improve resilience in older adults in an important setting: senior housing communities. |
| 41 | Quinn et al.  (2009) | 2009 | UK | Mixed methodology approach | Mental Health (Aging attitude) | Positive attitudes to ageing predicted stronger endorsement of health-related behaviors, while negative attitudes to ageing and mental illness predicted lower subjective well-being. |
| 42 | Bryant et al.  (2012) | 2012 | UK | Cross-sectional postal survey | Mental Health (Aging attitude) | Having positive attitudes to aging may contribute to healthier mental and physical outcomes in older adults. |
| 43 | Deshayes et al.  (2021) | 2021 | France | Empirical research | Mental Health (Aging attitude) | The associations between personality traits and physical capacities are different according to the physical capacities investigated. |
| 44 | Nakamura et al.  (2022) | 2022 | USA | Cohort study | Mental Health (Aging attitude) | In the highest (vs lowest) quartile of aging satisfaction was associated with improvements in some health behaviors (eg, increased likelihood of engaging in frequent physical activity), physical health conditions (eg, reduced risk of mortality), and psycho-social well-being factors (eg, reduced risk of depression) 4 years later, conditional on pre-baseline aging satisfaction. |
| 45 | Hedberg et al.  (2010) | 2010 | Sweden | Cross-sectional study | Mental Health (Meaning in life) | The very old people studied were feeling indecisive about their purpose in life and that feelings are linked with poorer psychological health. |
| 46 | Ness et al.  (2014) | 2014 | Norway | Narrative interview | Mental Health (Meaning in life) | Despite being at a critical stage of life, these older individuals were able to draw on inner resources to maintain a positive outlook on physical decline, loneliness, and various life adversities, and to pursue new meaning in the challenges of aging. |
| 47 | Heisel and Flett  (2016) | 2016 | Canada | Longitudinal study | Mental Health (Meaning in life) | MIL may play an important role in promoting mental health and well-being and potentially conferring resiliency to contemplation of suicide in later life. |
| 48 | Czekierda et al.  (2017) | 2017 | USA | Systematic review and meta-Analysis | Mental Health (Meaning in life) | The strongest associations were found for subjective indicators of physical health. |
| 49 | Chen et al.  (2022) | 2022 | China | Cross-sectional study | Mental Health (Meaning in life) | Meaning in life promotes mental health through three pathways of attitudes toward aging, namely positive attitudes toward physical changes, psychological growth, and psycho-social loss, although the underlying mechanisms differ. |
| 50 | Nie et al.  (2023) | 2023 | China | Meta-analysis | Mental Health (Meaning in life) | This finding identify a moderate negative correlation between meaning in life and mental health issues among older adults. |
| 51 | Huxhold et al.  (2014) | 2014 | Germany | Longitudinal study | Social support  (Informal Social Activities) | Social activities differentially affect different facets of well-being. These associations change with age. In older adults, the effects of social activities with friends may become more important and may act as a buffer against negative effects of aging. |
| 52 | Tao and Shen  (2014) | 2014 | China | Field survey | Social support  (Informal social support) | Receiving financial support,daily care from adult children has positive effect on the physical and mental health of the elderly. The informal social support from non-core relatives and friends of the elder also play a beneficial role in "main-effect model" while the formal support make a "buffer model" effect. |
| 53 | Cheng  (2016) | 2016 | China | Empirical research | Social support  (Informal social support) | As family size continues to shrink and become more nuclear, co-residence becomes increasingly difficult to achieve and family support gradually weakens, leading to a growing demand among older adults for social support and institutional eldercare services. |
| 54 | Sun et al.  (2016) | 2016 | China | Empirical research | Social support  (Social capital) | Both individual social capital and community social capital have significant positive effects on self-rated health, with community social capital exerting a stronger health-promoting effect. However, due to the underdevelopment of public service infrastructure in rural areas and the separation between individuals and communities in urban settings characterized by more developed social networks, there is a lack of a foundation for health-promoting interactions between individuals and communities. |
| 55 | Liu et al.  (2017) | 2017 | China | Empirical research | Social support  (Formal social support) | The medical insurance system plays a crucial role in improving older adults’ self-rated and physical health, while the pension insurance system contributes to enhancing their mental and physical health. |
| 56 | Nieboer and Cramm  (2018) | 2018 | the Netherlands | Empirical research | Social support  (Informal social support) | Levels of age-friendliness and older people's ability to realize the instrumental goals to achieve overall well-being varied tremendously among neighborhoods, with older people living in less age-friendly communities reporting lower levels of well-being. |
| 57 | Zhou et al.  (2018) | 2018 | China | Empirical research | Social support  (Formal social support) | The receipt of pensions from the New Rural Pension Scheme significantly improves the mental health of rural older adults, particularly among rural women and individuals with lower socioeconomic status, whereas the corresponding effect of the New Rural Cooperative Medical Scheme is largely insignificant. |
| 58 | Iwagami and Tamiya  (2019) | 2019 | Japan | Opinion | Social support  (Formal social support) | The practical benefits in kind under the Long-Term Care Insurance system for family caregivers have been demonstrated. |
| 59 | Li et al.  (2022) | 2022 | China | Empirical research | Social support  (Formal social support) | Medical insurance has a significant positive effect on the mental health of middle-aged and older adults in rural areas, particularly among vulnerable groups, and this effect is especially pronounced among men, individuals aged 60 and above, and low-income populations. |
| 60 | Chi and Han  (2022) | 2022 | China | Field survey | Social support | Rural older people place greater importance on the quantity of formal social support, while urban older people place greater importance on the quality of formal social support. |
| 61 | Agerholm et al.  (2023) | 2023 | Northern Europe | Qualitative comparative study | Social support  (Formal social support) | There were differences in the organizational structure of the two care systems (nurses in hospitals and nurses in home healthcare) in relation to the integration between different actors and in accessibility to patient information, which influenced coordination. |
| 62 | Sugihara et al.  (2008) | 2008 | Japan | Longitudinal study (Panel study) | Social participation  (Productive Roles) | For women, none of the productive roles were found to be independently linked with depressive symptoms. Engaging in multiple productive roles, in comparison with doing only housework, was related to fewer depressive symptoms. |
| 63 | Levasseur et al.  (2010) | 2010 | Canada | Review | Social environment  (Social participation) | Depending on the main goal of these social activities, six proximal to distal levels of involvement of the individual with others were identified: 1) doing an activity in preparation for connecting with others, 2) being with others, 3) interacting with others without doing a specific activity with them, 4) doing an activity with others, 5) helping others, and 6) contributing to society. |
| 64 | Wang  (2011) | 2011 | China | Review | Social environment  (Social participation) | Older adults’ active participation in social and economic development is an effective way to address population aging. |
| 65 | Yu et al.  (2016) | 2016 | China | Empirical research | Social participation  (Re-employment) | Although the practice of “raising children for old-age support” still exists, the number of children an older adult has is positively associated with the probability of their children being employed, and it is also positively associated with net intergenerational transfers from parents to children. |
| 66 | Fu et al.  (2017) | 2017 | Japan | Longitudinal study | Social participation  (Social network) | Involvement in social networks was especially important for improving mental health among people with psychological distress. |
| 67 | Sala et al.  (2019) | 2019 | Japan | Empirical research | Social participation (Leisure activities) | Active engagement in leisure activities can help older adults to maintain cognitive, physical, and mental health. |
| 68 | Dehi and Mohammadi  (2020) | 2020 | Iran | Concept analysis | Social environment  (Social participation) | The defining attributes of the concept of elderly people’s social participation included emphasis on community-based activities and interpersonal interactions, based on resource sharing, active participation and individual satisfaction. |
| 69 | Du  (2022) | 2022 | China | Empirical research | Social environment  (Social participation type) | Leisure-based social participation among older adults has a statistically significant effect on both physical and mental health at the 1% level. In addition, participation in volunteer-based social activities has a significant effect on activities of daily living at the 10% significance level, a significant effect on instrumental activities of daily living at the 1% level, and a significant effect on depression at the 5% level. |
| 70 | Fang et al.  (2020) | 2020 | China | Empirical research | Social participation (Volunteer service participation) | Volunteer service participation significantly improves residents’ happiness, and this effect is robust to sample heterogeneity. It enhances social skills, trust, and perceived fairness, thereby increasing happiness. However, volunteering organized by government agencies shows no significant effect, while participation through social organizations or multiple channels has a significant positive impact. |
| 71 | Fain et al.  (2022) | 2022 | UK | Cohort study | Social environment  (Social participation) | This study's findings provide evidence of an association between social participation and lower all-cause mortality for older adults. They also suggest that the effect of social participation on health is greater for people who are more physically active. |
| 72 | Zhou et al.  (2023) | 2023 | China | Longitudinal study | Social participation (Self-employment) | Self-employment can significantly reduce the depression tendency of the younger elderly and promote their mental health. self-employment can indirectly improve the mental health of the younger elderly through income growth effect and self-worth realization effect, in which the self-worth realization effect is greater than the economic effect. |
| 73 | Alley et al.  (2007) | 2007 | USA | Review | Physical environment  (Age-friendly cities) | This paper presents an analysis of the literature and results of a Delphi study identifying the most important characteristics of an elder-friendly community: accessible and affordable transportation, housing, health care, safety, and community involvement opportunities. |
| 74 | Scharlach  (2012) | 2012 | USA | Review | Physical environment  (Aging-friendly communities) | This article describes the types of community aging-friendly initiatives that currently exist in the United States, and the roles that various sectors (e.g., public, non-profit, private) have played in their development. |
| 75 | Rooney et al.  (2013) | 2013 | UK | Review | Physical environment  (Age-friendly cities) | The idea of "Lifetime Homes," encompassing housing, free transportation, green spaces, and community safety.The standards should be improved and that designers and architects face challenges to creatively incorporate them into housing design |
| 76 | Fitzgerald and Caro (2014) | 2014 | USA | Introduction | Age-friendly Cities and Communities | To the extent that they may have limited access to own transport, older people and children share an interest in good pedestrian features and public transportation. They also share interest in local parks but differ in the park features of interest. |
| 77 | Gao et al.  (2016) | 2016 | USA | Cross-sectional study | Built environment | In the new neighborhoods, increased perceived diversity, safety, and esthetic were significantly associated with higher physical and mental well-being. |
| 78 | Choi  (2020) | 2020 | China | Empirical research | Physical environment  (Age-friendly communities) | Both a greater perceived availability of age-friendly features in communities and a good person-environment fit were associated with better self-rated health and a lower likelihood of reporting functional limitations. |
| 79 | Cerletti et al.  (2021) | 2021 | Switzerland | Cohort study | Built environment | Higher health-related quality of life and less health care utilization were associated with less reported transportation noise annoyance. Higher health-related quality of life was also associated with greater satisfaction with the living environment and more perceived access to greenspaces. |
| 80 | Torku et al.  (2021) | 2021 | USA, Canada, UK and Hong Kong | Systematic Review | Age-friendly Cities and Communities | The article highlighted important issues that the need to conduct collaborative research among developed and developing countries through crossnation comparisons; the need to perceive older adults as place-makers and value resources in Age-friendly Cities and Communities; the need to conduct a prior participatory analysis to identify appropriate participatory measures that can maximize the participation of older adults in the development of age-friendly cities and communities. |
| 81 | Wang et al.  (2023) | 2023 | China | Scoping Review | Built Environment and Social Environment | Built and social environments influence physical activity, and consideration of people’s perceptions of their surroundings can provide further insight. |
| 82 | Pan et al.  (2024) | 2024 | China | Longitudinal study | Age-friendly neighbourhood environment | The age-friendliness of transportation was positively associated with functional abilities, and housing was the primary factor that shaped life satisfaction at the baseline. Better maintenance of functional abilities could promote life satisfaction over time. |
| 83 | Zhang and Yang  (2024) | 2024 | China | Systematic review | Physical environment  (Public transport inclusion) | Transport inclusion can help enhance elderly health, social participation, and subjective well-being. It can improve their travel opportunities for active aging in four ways: opportunity accessibility, physical accessibility, age-friendly informationalization, and cost affordability. |
| 84 | Tang et al.  (2025) | 2025 | China | Empirical research | Physical environment  (Age-friendly communities) | Physical health was mainly positively influenced by green spaces and negatively affected by public healthcare services; apart from public transportation, most age-friendly communities components were indicative of psychological health; and social networks were primarily influenced by green spaces and community support. |
| 85 | Byrnes et al.  (2006) | 2006 | USA | Empirical research | Physical environment  (Home modifications) | Home hazards, neighborhood hazards, geographic location, and interaction press measures predicted housing satisfaction and neighborhood satisfaction. Respondents who reported the lowest levels of mental and physical functioning also reported the lowest levels of residential satisfaction and faced the greatest environmental challenges. |
| 86 | Petersson et al.  (2008) | 2008 | USA | Empirical research | Physical environment  (Home modifications) | Home modifications have a positive impact on self-rated ability in everyday life, especially on decreasing the level of difficulty and increasing safety. |
| 87 | Wiseman et al.  (2021) | 2021 | USA | Empirical research (A cross-sectional study) | Physical environment  (Home modifications) | Home modifications are a promising tool to reduce falls and fall-related injuries in older adults. |
| 88 | Che et al.  (2024) | 2024 | China | Empirical research (A cross-sectional study) | Physical environment  (Home modifications) | Perception of age-friendliness of a city is associated with life satisfaction, and the effect of perception of age-friendliness on life satisfaction is partially mediated by attitudes to aging. |
| 89 | Lyu et al.  (2025) | 2025 | China | Empirical research | Physical environment  (Home modifications) | Positive associations between age-friendly home modifications and both self-rated health and life satisfaction. Attitudes towards aging were observed to mediate the relationship between home modifications and well-being, with living alone and internet use acting as positive moderators, while age served as a negative moderator. |
| 90 | Vasara  (2015) | 2015 | Finland | Narrative analysis | Assistive tech  (Aging in place) | These narratives support studies emphasizing the importance of home in regard to well-being. |
| 91 | Fernández-Carro  (2016) | 2016 | Spain | Empirical research | Assistive tech  (Preferred care and residential arrangements) | Residential preferences vary depending on expected health conditions. Remaining in one’s own home is preferred when older people foresee a healthy old age, whilst co-residence at a relative’s home turns into the favoured solution if older people have to face some physical or cognitive limitation. |
| 92 | Khosravi and Ghapanchi  (2016) | 2016 | Australia | Systematic review | Technological environment (Assistive tech) | Assistive technologies that have been proposed to overcome dependent living, fall risk, chronic disease, dementia, social isolation, depression, poor well-being, and poor medication management. This finding categorized these assistive technologies into six clusters, namely, general information and communication technology,robotics, telemedicine, sensor technology, medication management applications, and video games. |
| 93 | Calvaresi et al.  (2017) | 2017 | Italy | Systematic review | Assistive tech  (the ambient assisted living domain) | There is a lack of concrete evaluation concerning the actual usability, effectiveness and efficiency of the proposed solutions in achieving the emerging needs. |
| 94 | Mostaghel and Oghazi  (2017) | 2017 | Sweden | Empirical research (Qualitative comparative analysis) | Technological environment (Assistive tech) | The necessary conditions for high "perceived ease of use" and "perceived usefulness" are gerontechnology self-efficacy, gerontechnology anxiety, and cognitive abilities |
| 95 | O’Brolcháin  (2018) | 2018 | Ireland | Perspective article | Technological environment (Assistive tech) | Assistive technologies are not necessarily beneficial in terms of autonomy and examine ways that they might be used to undermine the autonomy of Intellectual and Developmental Disabilities, specifically the categories of knowledge, authenticity, and liberty. |
| 96 | Van Hoof and Marston  (2021) | 2021 | The Netherlands | Editorial | Technological environment (Assistive tech) | The body of knowledge presented here in this special issue acknowledges the importance of the interplay surrounding ageing, urbanization, and digitization (technology). |
| 97 | Sehgal et al.  (2021) | 2021 | USA | Review | Technological environment (Mobility Assistive Device) | Many individuals need a mobility assistive device as they age. These devices include canes, crutches, walkers, and wheelchairs. Clinicians should understand how to select the appropriate device and size for individual patients (or work with a physical therapist) and prescribe the device using the patient's health insurance plan. |
| 98 | Bergschöld et al. (2024) | 2024 | Norway | Scoping Review | Technological environment (Technologies for Aging in Place) | Redundancies and unexploited synergies between bodies of evidence on technology for aging in place are highly likely. |
| 99 | Ordonez et al.  (2011) | 2011 | Brazil | Empirical research | Technological environment (Digital inclusion) | Digital inclusion can represent an important strategy for cognitive enhancement for older adults, which may assist them to continue to perform daily activities independently. It also proposes that computer use can also contribute to their physical and mental health and to their quality of life. |
| 100 | Winstead et al.  (2013) | 2013 | USA | Empirical research (Qualitative analysis) | Technological environment (Digital inclusion) | Information and communication technology have the potential to allow individuals to transcend social and spatial barriers, providing residents with the ability to maintain and enhance social networks as well as provide a greater sense of connection to the world at large. |
| 101 | Bobillier Chaumon et al.  (2014) | 2014 | France | Empirical research | Technological environment (Digital inclusion) | Information and communications technologies may, to some extent, play an instrumental role in interconnectedness and social stimulation, and can also be seen as a 'boundary object' that communicates between the residents’ world (who are rather isolated) and their families’ world (including grandchildren). |
| 102 | Wong et al.  (2014) | 2014 | China | Empirical research | Technological environment (Digital inclusion) | Learning how to use a computer improved some aspects of the self-efficacy of the participants, especially in areas related to the handling of computers, and heightened their awareness that being unfamiliar with information and communication technology would result in marginalisation. |
| 103 | French and Richardson  (2017) | 2017 | UK | Empirical research | Technological environment (Digital inclusion) | The effectiveness of embedding digital inclusion support within wider social inclusion services at the local level, mediated by institutions and organization which have established pathways to engage socially excluded people, including disabled people. |
| 104 | Fisk et al.  (2023) | 2023 | USA | Empirical research | Technological environment (Digital inclusion) | This finding highlights the importance of collaborative integration of service provider expertise and service customer lived experiences—both of which have value for facilitating digital inclusion. |
| 105 | Golant  (2017) | 2017 | USA | Theoretical research | Technological environment (Smart elderly care) | Smart technology products have considerable potential as solutions that enable older persons to experience more comfortable and independent lives in their current homes and communities. |
| 106 | Pal et al.  (2019) | 2019 | Thailand | Empirical research | Smart elderly care (Smart-Home Revolution) | A negative perception modeling has been used for this work to identify the factors that resist the Internet of Things based smart-home adoption among the elderly people in an Asian context. Two constructs (perceived-uselessness and self-efficacy) were found to be non-significant, while the remaining proved to be significant (innovativeness,perceived-reliability,perceived-interoperability,service-cost,privacy-concern,psychological-barrier, home administrative policy, and government-policy). |
| 107 | Park et al.  (2017) | 2017 | Korea | Longitudinal study | Person-environment fit perspective | Among low-income individuals, the supportive environment of senior housing plays a pronounced compensating role and may be a key to successful adaptation in aging. |
| 108 | Park and Lee  (2017) | 2017 | Korea | Empirical research | Person-environment fit perspective | Age-friendly environmental contexts may have both benefits and detriments for vulnerable older adults' well-being, depending on P-E fit. |
| 109 | Choi  (2020) | 2020 | China | Empirical research | Person-environment fit perspective | Both a greater perceived availability of age-friendly features in communities and a good person-environment fit were associated with better self-rated health and a lower likelihood of reporting functional limitations. |
| 110 | Hewston et al.  (2021) | 2021 | Canada | not mention | Person-environment fit perspective | Within the PEO model, life-space is an environmental construct that can influence occupational performance in older adults. The framework outlined in this paper extends the focus of frailty beyond the person factors to encompass the environmental context in which occupations occur. |
| 111 | Fang et al.  (2023) | 2023 | China | Cross-sectional study | Person-environment fit perspective | The present findings offer crucial evidence for understanding the interactions between a person and the environment, as well as their influence on physical activities of daily livings, suggesting the importance of a supportive environment and a subpopulation-targeting strategy for disabled older adults |
| 112 | Liu et al.  (2023) | 2023 | China | Empirical research | Person-environment fit perspective | Community environmental resources (basic infrastructure and voluntary organizations) had a protective effect on frailty development and buffered the negative effects of SES vulnerability experiences accumulated over the life course. Community basic infrastructure resources played an important role in slowing the progression of frailty for individuals with cumulative SES disadvantage and downward mobility. |
